# “*Euphoria*” or “*Only Teardrops*”? Eurovision Song Contest performance, life satisfaction and suicide

**DOI:** 10.1186/s12889-018-5497-3

**Published:** 2018-05-11

**Authors:** Filippos T. Filippidis, Anthony A. Laverty

**Affiliations:** 1 Department of Primary Care and Public Health, School of Public Health, Imperial College London, 310 Reynolds Building, St. Dunstan’s Road, London, W6 8RP UK; 2 Public Health Policy Evaluation Unit, School of Public Health, Imperial College London, 310 Reynolds Building, St. Dunstan’s Road, London, W6 8RP UK

## Abstract

**Background:**

The popularity of the Eurovision Song Contest (ESC) in Europe has been high for decades. We aimed to assess whether a country’s performance in the ESC is associated with life satisfaction and suicide mortality in European countries.

**Methods:**

We analysed nationally representative Eurobarometer survey data on life satisfaction from 33 European countries (*N* = 162,773) and country-level standardised suicide mortality data for years 2009 to 2015. The associations of winning the Contest, performing terribly, and higher final ranking with life satisfaction and suicide rates were all assessed.

**Results:**

Winning the ESC was not statistically significantly associated with increased life satisfaction or suicide rates, although every ten-place increase in final ranking was associated with an increase in life satisfaction (adjusted odds ratios [aOR] 1.04; 95% confidence interval [CI]: 1.02 to 1.05) and a decrease in suicide mortality rates (β = − 0.30; 95% CI: -0.59 to − 0.01). Terrible performance was associated with greater life satisfaction compared to not competing at all (aOR 1.13; 95%CI: 1.07 to 1.20).

**Conclusion:**

The good news for participating countries is that just competing at the ESC is associated with higher life satisfaction among the population. As improved performance is linked to *Ooh Aah Just a Little Bit* of improved life satisfaction, further research into how such international competitions may impact public health is needed.

## Background

The annual Eurovision Song Contest (ESC, hereafter “The Contest”) is a seminal event for both music lovers and haters everywhere. Established in 1956, is one of the longest-running TV shows in the world, and firmly embedded in the consciousness of populations worldwide, especially in Europe [1]. Each participating country’s entry is ranked by a combination of votes from national juries and audience. The format of the Contest changed in 2004 and semi-finals were introduced in order to accommodate the increased number of participating countries. Some countries are thus eliminated in the semi-finals, while the remainder (usually 24–27 countries) join the large countries which have a guaranteed place in the final thanks to their large monetary contributions to the European Broadcasting Union (EBU) [1]. Each country awards points to the ten highest ranked entries giving the famous “douze points” (i.e. 12 points) to the entry that receives the most votes. Some viewers, such as the British, are more familiar with the infamous, albeit grammatically troublesome, “nil points”.

The contest is very popular in Europe. EBU, organisers of the event, announced that the 2016 shows reached a total of 204 million viewers across 42 markets, with audience shares as high as 95.3% -in music-loving Iceland, where almost *Everybody (entry of Estonia 2001)* was watching [2]. The Contest attracts a wide range of individual countries’ talent, including orc-style monsters (Finland 2006), singers on unicycles (Moldova 2011) and puppet turkeys (Ireland 2008). Entries often feature unusual lyrics such as “*Boom boom boomerang, snadderydang, kangaroo, boogaloo, didgeridoo”* (Austria, 1977) and “*I look over all the maps trying to escape ‘cause I’m tired of your sweet cheesecake”* (Belarus, 2014), which is considered part of the ESC charm. Not surprisingly, the ESC is considered a seminal event in certain countries and among some population groups with winners being hailed as *Heroes (Sweden 2015)*; the majority of the population is aware of the contest, even if they do not watch it live [3]. The Contest has recently expanded to other continents; Australia has been participating since 2015.

Large national and international competitions which attract a lot of interest among the general population may have unanticipated consequences. The associations between major sporting events and suicides and homicides, [4] reported happiness in the general public, [4, 5] and cardiovascular events [6] have been researched, although the causal nature of these associations is debatable. The ESC, beyond being a major international event, is further influenced by political and socioeconomic factors, [3, 7] therefore it is reasonable to consider that it may have a similar impact. However, the literature on the potential impact of international music events on such outcomes is limited; to the authors’ knowledge, no study has assessed the association between ESC -or any other similar music event- and life satisfaction, mental health or suicide. A potential association with specific diseases would have obvious implications for public health, but the same can be argued for outcomes such as life satisfaction. Life satisfaction has been linked with both mental disorders and physical health, mortality and morbidity [8–13]. Therefore it can serve as a general indicator of population health. Considering the popularity and perceptions of importance of the ESC among the European population, [3] we hypothesized that performance in the ESC may have an impact on life satisfaction and mental health.

## Methods

### Data sources and measures

Individual level data were collected through a series of waves of the Eurobarometer survey which covers European Union (EU) member states and occasionally other European countries [14]. We used waves that included a question on life satisfaction and were conducted in the period immediately after the ESC finals, i.e. May–June of the respective year. The total sample size of the repeated cross-sectional waves 71.2 (2009), 73.5 (2010), 75.4 (2011), 77.4 (2012), 79.4 (2013), 81.4 (2014) and 83.4 (2015) was 198,008 individuals from 33 countries (all 28 EU member states, Turkey, the Former Yugoslavian Republic of Macedonia, Serbia, Montenegro and Iceland).

The multi-stage sampling methodology was consistent across all waves. Primary sampling units (PSU) were selected from each region of each country, proportional to population size. In each PSU, a sample of starting addresses was randomly selected, and households were systematically selected through a standard random route starting from these addresses. Post-stratification and population size weighting were applied, resulting in samples aged ≥15 years nationally representative in terms of age, sex and area of residence [15]. All data is self-reported and collected through face-to-face interviews. Participants in each survey wave are independently sampled, which means that there is no longitudinal sample across multiple years.

Life satisfaction was assessed with the question “On the whole, are you very satisfied, fairly satisfied, not very satisfied or not at all satisfied with the life you lead?” Responses were grouped into “very satisfied” and “not very satisfied” (fairly satisfied; not very; or not at all satisfied). Eurobarometer surveys also collected data on participants’ age (15–24; 25–34; 35–44; 45–54; 55–64; and ≥ 65 years), sex (male; female), occupation (employed; houseperson; student; unemployed; and retired), age at which they stopped full-time education: (≤15; 16–19 and ≥ 20 years old), marital status (married or living with partner; single; divorced; widowed; and other), area of residence (urban; rural) and their difficulties to pay bills during the last 12 months (almost never/never; and from time to time/most of the time).

Country-level data for years 2009 to 2015 were extracted from the World Health Organization’s (WHO) European Health for all Database (HFA-DB) [16]. We extracted data on real gross domestic product (GDP Purchasing Power Parity in US dollars [PPP$] per capita); total health expenditure (PPP$ per capita); unemployment (%) as a proportion of the active population; as well as age-standardized death rate from suicide and self-inflicted injury (SDR per 100,000 population) in the entire population and separately among males and females.

Data on each country’s performance in the ESC were extracted from the webpage www.EurovisionWorld.com which includes a fan-made “complete database of all the songs, lyrics and votes from all the history of Eurovision” [17]. Since 2004, when the semi-finals were introduced, 24–27 countries compete in the final. For these countries, we recorded their final rank. Countries which did not qualify from the semi-finals were all assigned one place lower than the last contestant in the final; for example, if the final consisted of 25 countries, all eliminated countries were considered to have been ranked 26th. Rank was scaled to show results for a ten-unit increase. Finally, contestants that took the 20th or lower place were classified as having had a “terrible” performance on that year.

### Statistical analyses

We conducted two analyses using life satisfaction as an outcome: first to assess the impact of final performance and winning, and second to assess whether terrible performance was better than not competing at all.

A multilevel logistic regression model was fitted, with country as the higher level of analysis. This model accounts for clustering of observations within each country and hence allows us to explore changes in life satisfaction within countries over the time period we have studied. Being very satisfied with the life they lead was the outcome variable. Independent variables included final rank in the ESC and being a winner of the contest, adjusted for GDP per capita, unemployment rate in the country, age, sex, area of residence, occupation, education, difficulty paying bills, marital status and a linear term for time (calendar year) to account for underlying trends in life satisfaction. We also stratified these analyses by sex – women have won the ESC more than twice as much as men, [18] which may be expected to result in differential effects between sexes.

A separate multilevel logistic regression was fitted in the subset of observations (i.e. country-years) that did not participate in the contest or performed terribly, to compare the potential effect of a terrible performance in ESC relative to not participating at all. Life satisfaction was the outcome and a variable for terrible performance (versus no participation) was the main independent variable; the model was adjusted for the same variables as above.

We also conducted ecological analyses of country-level suicide rates to assess if potential associations hold for mental health indicators as well. A longitudinal fixed-effects linear regression model was fitted to capture potential associations of success in the ESC with standardised suicide mortality in the total population and separately among males and females within each country. The fixed-effects specification controls for country-level unobserved factors that could be associated with independent variables, which allows us to estimate associated changes between independent and outcome variables within countries over time, but not between-country associations. The analysis was conducted at a country level and independent variables included final rank in the ESC, being a winner of the contest, GDP per capita, health expenditure per capita, and unemployment. A linear variable for time (calendar year) was also included in the model. Separate models were used to assess terrible performance versus not competing.

## Results

After excluding observations from countries and years that did not participate in the ESC, we had individual level data for *n* = 162,773 respondents from 33 countries. Winning the contest was not significantly associated with self-reported life satisfaction (adjusted odds ratio [aOR] = 1.05; 95% Confidence Interval [CI]: 0.98 to 1.12). However, the aOR of reporting high life satisfaction was 1.04 (95% CI: 1.02 to 1.05) for every 10 places of better performance in the ESC among the entire sample, controlling for time, sociodemographic and macro-economic factors (Table 1). When we stratified by gender, results were very similar. Having performed terribly in the ESC (*n* = 115,520) was associated with higher odds of reporting high life satisfaction compared to not participating in the contest at all (aOR = 1.13; 95% CI: 1.07 to 1.20), a finding that was consistent among both men and women (Table 2).

**Table 1:** Fully adjusted associations between Eurovision Song Contest winning and final rank performance and life satisfaction in Europe

	Total population aOR (95% CI)	Males aOR (95% CI)	Females aOR (95% CI)
Winning the ESC			
No (ref)	1.00	1.00	1.00
Yes	1.05 (0.98 to 1.12)	1.06 (0.96 to 1.17)	1.04 (0.95 to 1.15)
Higher rank in the ESC (per 10 places)	*1.04 (1.02 to 1.05)*	*1.04 (1.01 to 1.07)*	*1.03 (1.01 to 1.06)*

Models adjusted for sex, age, marital status, occupation, education, area of residence, difficulty paying bills, GDP per capita, unemployment, time and all factors included in the table

Results in italics indicate statistically significant results at the 0.05 level

**Table 2:** Fully adjusted associations between terrible performance in the Eurovision Song Contest and life satisfaction in Europe

	Total population aOR (95% CI)	Males aOR (95% CI)	Females aOR (95% CI)
Performing terribly			
No participation (ref)	1.00	1.00	1.00
Being ranked 20th or worse	*1.13 (1.07 to 1.20)*	*1.14 (1.04 to 1.25)*	*1.13 (1.04 to 1.22)*

Models adjusted for sex, age, marital status, occupation, education, area of residence, difficulty paying bills, GDP per capita, unemployment, time and all factors included in the table

Results in italics indicate statistically significant results at the 0.05 level

Our ecological analyses had country-level data for 43 countries of the European region of the WHO, which participated at least once in the ESC between 2009 and 2015. The final rank in the ESC was associated with lower SDR (per 100,000) from suicide and self-inflicted injury in both the total population (β = − 0.30; 95% CI: -0.59 to − 0.01 per ten places) and males (β = − 0.54; 95% CI: -1.05 to − 0.37), but not in females (β = − 0.09; 95% CI: -0.24 to 0.06) (Table 3). On the contrary, winning the ESC was not statistically significantly associated with SDR from suicide. When comparing countries that performed terribly with those that did not participate at all (Table 4), there was no statistically significant difference in SDR from suicide.

**Table 3:** Association between Eurovision Song Contest winning and final rank and standardized death rate (SDR) from suicide and self-inflicted injury (*n* = 175)

	SDR suicide in total population β (95% CI)	SDR suicide in males β (95% CI)	SDR suicide in females β (95% CI)
Winning the ESC	0.37 (−0.98 to 1.72)	0.62 (−1.74 to 2.99)	0.09 (− 0.63 to 0.81)
Higher rank in the ESC (per 10 places)	*−0.30 (− 0.59 to − 0.01)*	*−0.54 (− 1.05 to − 0.37)*	−0.09 (− 0.24 to 0.06)

Models adjusted for GDP per capita, health expenditure, unemployment, time and variables included in the table

Results in italics indicate statistically significant results at the 0.05 level

**Table 4:** Association between terrible performance in the Eurovision Song Contest and standardized death rate (SDR) from suicide and self-inflicted injury (*n* = 129)

	SDR suicide in total population	SDR suicide in males	SDR suicide in females
	β (95% CI)	β (95% CI)	β (95% CI)
Performing terribly (vs. not participating)	0.32 (− 1.15 to 0.51)	−0.40 (− 1.84 to 1.04)	− 0.31 (− 0.79 to 0.17)

Models adjusted for GDP per capita, health expenditure, unemployment, time and variables included in the table

## Discussion

We found that although winning the Eurovision Song Contest was not associated with improved life satisfaction or decreased mortality from suicide, a country’s overall performance was associated with *Ooh Aah Just a Little Bit (UK 1996)* of an improvement in life satisfaction and a decline in SDR from suicide. Better performance was associated with higher odds of being very satisfied in both men and women. Performing terribly in the Contest was associated with increased life satisfaction compared to not participating at all.

Ranking in the ESC may reflect the international position of a country within the world. It has been found that there are clusters of countries which exchange votes year after year, based on cultural, geographic, economic and political factors [19–21]. The Contest has even been studied as a “friendship” network [22]. Nevertheless, it is a complex and constantly changing network and it is hard to disentangle the factors influencing this [23, 24]. Thus, performance in the ESC might be a reflection of alliances with other countries, as well as of the political and economic conditions in the country. Therefore, ESC success could be a proxy of favourable socioeconomic conditions, which in turn can create *Euphoria (Sweden 2012)* and positively influence life satisfaction and mental health. Eurovision is a stage where representations of the participating nations can reach a wide international audience [7] hence the performance in the contest may be perceived as a judgment –by an international audience- of nationally defining characteristics, which are commonly part of the performance, even if they are sometimes hidden behind glitter and cheeky *Wild Dances (Ukraine 2004)*. A large proportion of the ESC audience *Believe (Russia 2008)* that voting has at least some geopolitical element [3]. Consequently, ESC success may be perceived as a sign of a country’s strength at the international stage.

On the other hand, doing well in competitions might increase productivity among the supporters of the contestants. For example, there is some evidence that both productivity and per capita income increase in the host city of the winning team of the Super Bowl, one of the largest sports events in the world [25] and that winning the FIFA World Cup might be beneficial for tourism [26]. More than that, better than expected performance in major sports events may raise levels of happiness among the supporters [5]. If doing well in the ESC has a similar beneficial impact, it is not a surprise that, following a successful performance in the ESC, life satisfaction levels *Rise like a Phoenix (Austria 2014)*. In our study, being the *Number One (Greece 2005)* in the competition did not have an additional effect on neither suicides nor life satisfaction, which is consistent with findings from sports performance in the United States [4].

ESC songs get considerable airtime, especially around the time of the Contest. A good song can provide a moment of (*Hard Rock) Hallelujah (Finland 2006)* for people and improve their quality of life. However, songs performed in the Contest are not usually considered of high quality, which makes this explanation seem like a *Fairytale (Norway 2009)* to the authors. It is telling that only about one in five people watch the ESC because they like the actual music [3]. Additionally, the Contest only takes place once a year and hence the songs get limited airtime during the rest of the year.

We found that participating in the ESC and doing badly is better than not participating at all. Thus, there is no public health risk in taking part, as even an abysmal performance would be better than complete absence from the contest. This may be particularly important for the United Kingdom, where a “Eurovision Brexit” is gaining support in response to the country’s consistently terrible performances [3]. Our findings suggest that, if there is an underlying causal link between ESC performance and life satisfaction, in the unlikely event that the UK performs well in the ESC, life satisfaction in the country would significantly improve, but even being the *Cry Baby (UK 2003)* of the Contest has more public health benefits than *Running Scared (Azerbaijan 2011)* away from it. Life satisfaction has been associated with lower mortality and morbidity and has been found to have a reciprocal association with mental health problems [8, 11–13]. Such public health gains *Don’t Come Easy (Australia 2017)*, therefore Australia’s recent participation in the ESC or the money which the UK pays to the European Broadcasting Union to ensure a place in the final every year may represent good value for money for the public, although the cost-effectiveness of these investments should be further explored.

To our knowledge this is the first study to estimate the association between the ESC and life satisfaction. The availability of individual level data and the large sample size allowed us to adjust for potentially confounding factors; therefore, the authors believe that despite the cross-sectional design of the Eurobarometer surveys, they won’t meet their *Waterloo (Sweden 1974)* if they make a cautious attempt to hypothesize that there may be a causal association. However, the cross-sectional design of our study precludes any conclusions of causal nature. Even if there was a causal association, the fact that the surveys were conducted shortly after the ESC each year would only allow us to detect short-term effects. Also, we did not take into account the order of appearance; songs appearing towards the end are more likely to receive favourable evaluations [19, 27]. When exploring the association of ESC success with life satisfaction, we only considered country of residence, when it is an established fact that immigrants can play an important role in Eurovision voting, [21, 22] and therefore they might support and be influenced by entries from their home country. The prevalence of mental health disorders, including depression, varies among European countries, [28]; however, we used statistical approaches that account for such country-level variation. Finally, the ecological analysis is subject to ecological fallacy, even though the results were quite consistent between individual and country level analyses.

This study has quantified the potential benefits to life satisfaction and suicide mortality from performance in the ESC. Further research would be required to identify the *Secret Combination (Greece 2008)* of factors associated with improved performance. This may allow an informed discussion regarding the potential role of such issues in public health.

## Conclusion

The good news for any country entering the ESC is that it is not necessary to win to achieve improvements in the population’s life satisfaction; even competing at all is associated with higher life satisfaction among the population, while leaving can bring *Only Teardrops (Denmark 2013)*. Better performance seems to be linked to *Ooh Aah Just a Little Bit* of improved life satisfaction. Our findings highlight the potential association between popular events, such as the ESC, and outcomes of public health interest. Although more research into the topic is required to confirm this association and its nature, it is generally important to consider the unintended consequences of events that reach a wide audience. Mental health can be influenced by multiple factors, a complexity that is often ignored when considering changes at the population level. Our study shows that the meaning of Health-in-all-policies [29] may extend beyond what is normally considered public health domain.

## Abbreviations

aOR: Adjusted odds ratio
CI: Confidence interval
EBU: European broadcasting union
ESC: Eurovision Song Contest
EU: European Union
GDP: Gross domestic product
PPP: Purchasing Power Parity
PSU: Primary sampling unit
SDR: Standardized death rate
WHO: World Health Organization
